# New Indices of Endothelial Function Measured by Digital Thermal Monitoring of Vascular Reactivity: Data from 6084 Patients Registry

**DOI:** 10.1155/2016/1348028

**Published:** 2016-10-18

**Authors:** Morteza Naghavi, Albert A. Yen, Alex W. H. Lin, Hirofumi Tanaka, Stanley Kleis

**Affiliations:** ^1^MEDITEX, Houston, TX, USA; ^2^Endothelix, Inc., Houston, TX, USA; ^3^The University of Texas at Austin, Austin, TX, USA; ^4^University of Houston, Houston, TX, USA

## Abstract

*Background. *Endothelial function is viewed as a barometer of cardiovascular health and plays a central role in vascular reactivity. Several studies showed digital thermal monitoring (DTM) as a simple noninvasive method to measure vascular reactivity that is correlated with atherosclerosis risk factors and coronary artery disease.* Objectives. *To further evaluate the relations between patient characteristics and DTM indices in a large patient registry.* Methods. *DTM measures were correlated with age, sex, heart rate, and systolic and diastolic blood pressure in 6084 patients from 18 clinics.* Results. *DTM vascular reactivity index (VRI) was normally distributed and inversely correlated with age (*r* = −0.21, *p* < 0.0001). Thirteen percent of VRI tests were categorized as poor vascular reactivity (VRI < 1.0), 70 percent as intermediate (1.0 ≤ VRI < 2.0), and 17 percent as good (VRI ≥ 2.0). Poor VRI (<1.0) was noted in 6% of <50 y, 10% of 50–70 y, and 18% of ≥70 y. In multiple linear regression analyses, age, sex, and diastolic blood pressure were significant but weak predictors of VRI.* Conclusions. *As the largest database of finger-based vascular reactivity measurement, this report adds to prior findings that VRI is a meaningful physiological marker and reflects a high level of residual risk found in patients currently under care.

## 1. Introduction

Since the Framingham Heart Study first reported “risk factors” for atherosclerotic cardiovascular disease, numerous efforts have aimed at improving risk assessment in the asymptomatic population. Over time, these efforts have resulted in the introduction of Framingham Risk Score (FRS) and other biomarkers including the use of noninvasive imaging modalities such as coronary calcium scoring with CT scan and carotid IMT and plaque measurement. While these methods have shown prognostic values independent of risk factors, mainstream medicine is still relying on the FRS, which tends to be inaccurate for individualized risk assessment and fails to assess the current status of vascular health [1]. Moreover, FRS and risk factor-based scoring systems are neither designed nor used to monitor response to therapeutic interventions [2]. A comprehensive cardiovascular risk assessment requires measurement of risk factors as well as structural and functional markers of the arterial system. For the widespread acceptance and clinical adoption of a new test, it must be (1) incrementally predictive over risk factors, (2) responsive to therapy, (3) operator-independent and reproducible, (4) low-cost and widely accessible in primary care settings, and (5) posing no significant side effects. In recent years, endothelial function measurement has emerged as a reasonable candidate that could fit the above criteria. Endothelial function is viewed as a “barometer” of cardiovascular risk, and endothelial dysfunction is the “gateway” to atherosclerotic cardiovascular diseases [3, 4].

Over the past 20 years, a number of noninvasive methods of assessing peripheral endothelial function have been introduced, including ultrasound imaging of brachial flow-mediated dilatation (FMD), fingertip arterial tonometry, fingertip photoplethysmography, and laser Doppler flowmetry [5–12]. A new technique named digital thermal monitoring (DTM) has been developed to evaluate endothelial function by measuring vascular reactivity during a 5-minute arm-cuff reactive hyperemia test. DTM is the newest addition to the field and monitors fingertip temperature changes to measure vascular reactivity. DTM is a noninvasive, automated, and operator-independent test that can be performed both at physicians' offices and in patients' homes. We and other researchers have previously reported the relationships between DTM and CVD risk factors, coronary calcium score, myocardial perfusion defects, and coronary angiographic findings [13–20]. However, prior studies have been limited to a relatively small sample size. Therefore, the present study was conducted on a large registry of 6,084 patients from 18 different clinics to better characterize DTM index of vascular reactivity (VRI) in relation to patients' phenotypes.

## 2. Methods

**Figure 1:**
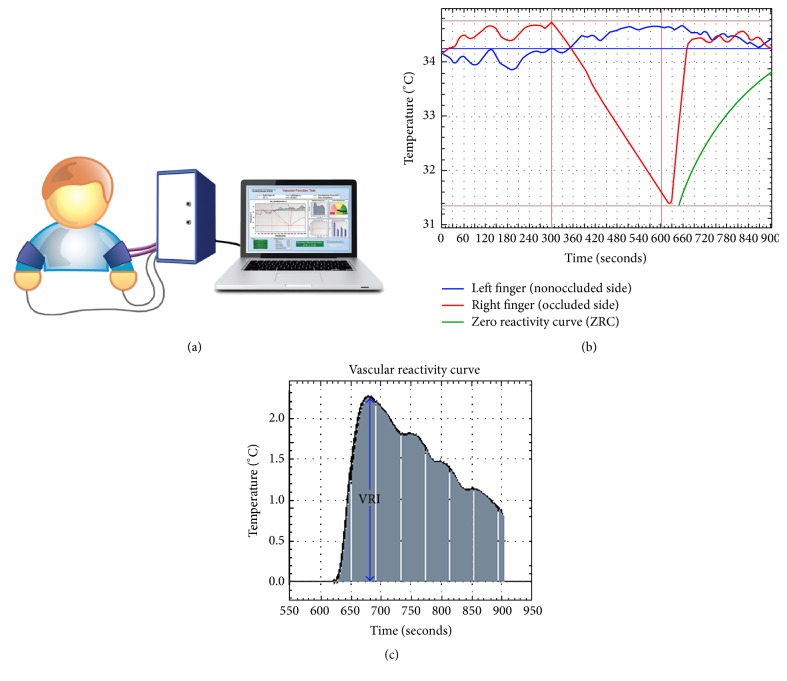
(a) Illustration of patient setup, with temperature sensors affixed to both index fingers and blood pressure cuffs on both arms. (b) A sample report screen displays a right finger temperature curve (red), a left finger temperature curve (blue), and a zero reactivity curve (green). (c) The software-generated, vascular reactivity curve is shown. The vascular reactivity index (VRI) is taken as the maximum value of this temperature curve during the reactive hyperemic period.

The methodology for measuring endothelial function and vascular reactivity using DTM has been previously described [21–25]. All DTM tests were performed using a VENDYS® 6000 Portable System (Endothelix, Houston, TX), a PC-based system that fully automates the cuff reactive hyperemia protocol. The general test setup and a sample VENDYS test report are shown in Figure 1. During subject preparation, blood pressure cuffs were placed on both of the subject's upper arms, and VENDYS skin temperature sensors were affixed to both of the subject's index fingers. The software-driven DTM test began with an automated measurement of blood pressure and heart rate obtained from the left arm cuff. Following a 5-minute period of patient and temperature stabilization, a 5-minute cuff occlusion (cuff inflated to 30 mmHg above systolic BP) of the right arm was performed. During the cuff occlusion period, fingertip temperature in the right hand decreased because of the absence of warm circulating blood. When the cuff was released after the 5-minute occlusion, hyperemic blood flow to the forearm and hand was restored, and this resulted in a “temperature rebound” in the fingertip that is directly related to the subject's hyperemic blood flow response, endothelial function, and vascular reactivity [21, 22]. Using the recorded fingertip temperatures, the ambient temperature of the testing room, the observed slope of temperature decline, and a multivariate bioheat formula, the VENDYS software calculated and plotted a zero reactivity curve (ZRC). The ZRC served as an internal control and showed the expected temperature rebound curve, if zero vascular reactivity was present and the other variables remained the same. In other words, the ZRC is the expected temperature curve, if no vasodilatation and subsequent reactive hyperemia had occurred [21]. Vascular reactivity index (VRI) was determined by taking the maximum difference between the observed temperature rebound curve and the ZRC during the reactive hyperemia period. VRI ranged from 0.0 to 3.5 and was classified as being indicative of poor (0.0 to <1.0), intermediate (1.0 to <2.0), or good (≥2.0) vascular reactivity.

The VENDYS DTM Test Registry includes age, sex, blood pressure, heart rate, VRI, and fingertip temperature measurements recorded during DTM tests. The Registry does not include other health related information. All DTM tests were performed in ambulatory care clinical settings. This study includes a total of 6,084 patients from 18 clinics that volunteered to submit their data to the Registry. The number of each type of medical practice is as follows: cardiology = 9, general/family practice = 4, antiaging = 3, and internal medicine = 2.

Statistical analyses were performed using MATLAB (The MathWorks, Inc., Natick, MA). Variable data were expressed as mean ± SD. VRI scores in men and women were compared using unpaired Student's *t*-test. Comparisons of categorical data (e.g., proportion of subjects with good VRI in men versus women) were performed using Fisher's exact test. Pairwise correlations were examined using Pearson's correlation coefficient, and correlations between VRI and multiple patient characteristics (i.e., age, sex, blood pressure, and heart rate) were evaluated using multiple linear regression analysis. *p* value < 0.05 was considered significant. When performing statistical comparisons, tests with missing data were excluded from the comparison. “Cold Finger Flag” was defined as the condition in which the right finger temperature at start of cuff occlusion (time 300 s) is ≤27°C. Previous DTM testing had shown that right finger *t*300 temperatures < 27°C often resulted in technically poor results. “Sympathetic Response Flag” was defined as the condition in which left finger temperature continuously declines (>0.5°C temperature drop over a 5-minute time period) after right arm-cuff occlusion. When evaluating VRI, tests that exhibited “Cold Finger Flag” (*n* = 353) or “Sympathetic Response Flag” (*n* = 294) were excluded from the analyses. In addition to monitoring temperature at the index finger of the right arm, we studied temperature changes at the index finger of the left (nonoccluded) arm and observed interesting signals that are currently under further investigations and not included in the results below.

## 3. Results

Selected patient and test characteristics are shown in Table 1. Overall, the study population had the typical age and sex distribution seen in internal medicine and cardiology clinics. Key characteristics included age 66 ± 14 yrs., 54% men, 46% women, systolic blood pressure (SBP) 138 ± 20 mmHg, diastolic blood pressure (DBP) 77 ± 12 mmHg, and heart rate (HR) 70 ± 13 bpm.

**Figure 2:**
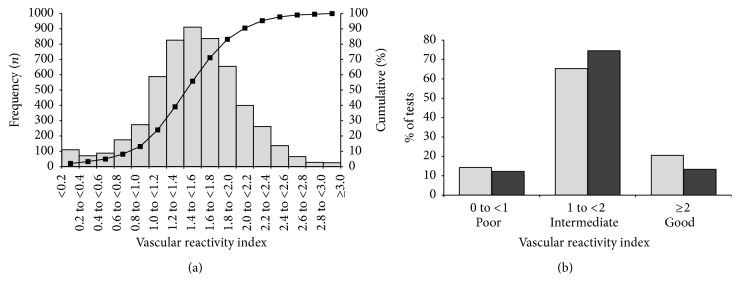
(a) Distribution of vascular reactivity index (VRI). A histogram and cumulative percentage curve are shown. (b) Distribution of vascular reactivity index (VRI) by gender. The percent of DTM tests falling into categories of poor, intermediate, and good vascular reactivity is shown for men (solid fill) and women (hatch fill).

The VRI distribution with cumulative percentages is shown in Figure 2(a). Overall, the VRI values exhibited the appearance of a normal distribution, with the exception of a small clustering of VRI values at or above zero. Thirteen percent of VRI tests were categorized as poor vascular reactivity (VRI < 1.0), 70% as intermediate (1.0 ≤ VRI < 2.0), and 17% as good (VRI ≥ 2.0). VRI was slightly higher in women than in men (1.56 ± 0.58 versus 1.50 ± 0.49; *p* = 0.0001). The distribution of poor, intermediate, and good VRI in men and women is shown in Figure 2(b). The percentage of good VRI was higher in women than in men (21% versus 13%; *p* < 0.0001). In contrast, men were slightly less likely to have poor VRI than women (12% versus 14%; *p* = 0.03).

**Figure 3:**
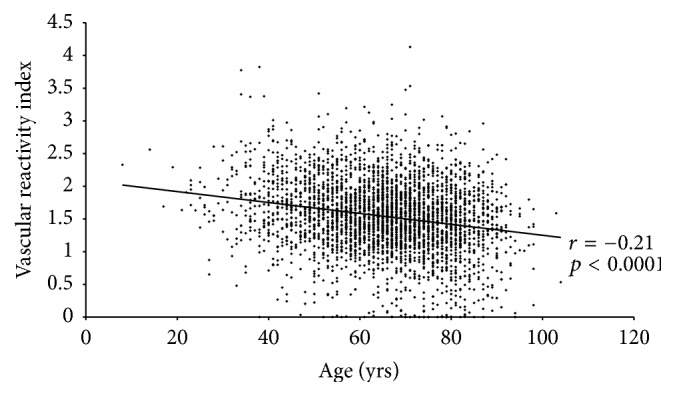
Vascular reactivity index (VRI) and age. A scatter plot, trend line, and Pearson's *r* coefficient are shown. VRI was mildly and inversely correlated with age.

**Figure 4:**
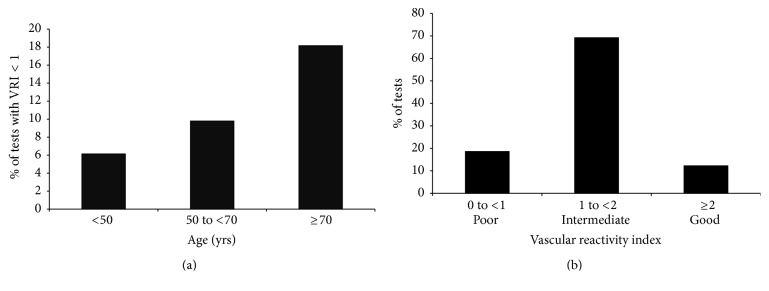
(a) Prevalence of poor VRI in different age groups. The frequency of having a poor VRI score (VRI < 1.0) is shown for the three age categories of age < 50 y, age 50–70 y, and age ≥ 70 y. (b) Distribution of vascular reactivity index (VRI) in oldest age group. The percent of tests falling into categories of poor, intermediate, and good vascular reactivity is shown for patients age ≥ 70 years.

VRI was mildly and inversely correlated with age (*r* = −0.21, *p* < 0.01) as illustrated in Figure 3. As shown in Figure 4(a), poor VRI (<1.0) was most frequent in the oldest age group (>70 yrs., 18%) compared with middle age (50–70 yrs., 10%) and younger (<50 yrs., 6%). However, the distribution of poor, intermediate, and good VRI values in this elderly age group (Figure 4(b)) was similar to that of the overall study population (13% poor, 70% intermediate, and 17% good).

VRI was not significantly correlated with SBP, DBP, pulse pressure (PP), or heart rate. A trend was seen of higher VRI scores in subjects with higher diastolic blood pressure (*r* = 0.10; *p* = NS). However, none of the blood pressure variables were significantly correlated with VRI.

Multiple regression models were built using VRI as the dependent variable and age, sex, SBP, DBP, and HR as independent variables. As shown in Table 2, age, sex, and diastolic blood pressure were significant but weak predictors of VRI.

## 4. Discussion

This is the largest report to date on any fingertip-based measurement of vascular reactivity and endothelial function [7, 26]. Our analyses showed that VRI values derived from DTM followed a near-normal distribution and the reasonable distribution conformed to previously established cutoff values for categorizing VRI scores as indicative of poor (0.0 to <1.0), intermediate (1.0 to <2.0), or good (≥2.0) vascular reactivity. VRI was weakly and inversely correlated with age. However, as shown in Figure 4(a), the frequency of poor VRI was three times higher in >70 y versus <50 y. This finding was in line with the findings of Framingham Heart Study reported by Hamburg et al. [26]. Nonetheless, as reported by Schnabel et al. in a community based study of 5,000 individuals, classical risk factors only accounted for 15.4% of FMD and 13.9% of PAT variability [7]. This clearly indicates that endothelial function provides a new angle into the status of vascular risk. The distribution of weak association between VRI and age is in sharp contrast to other vascular tests, including coronary artery calcium [27], carotid intimal-media thickness [28], and arterial stiffness [29], that are strongly and positively associated with age and, therefore, require age specific cutoffs. Although the highest prevalence of poor VRI was found in patients older than 70 y, the distribution of VRI values in this elderly population (Figure 4(b)) clearly shows a sizable number of good and intermediate scores. These findings support the clinical utility of DTM as a test that can differentiate good vascular function from poor vascular function, regardless of patient's age. We found VRI to be slightly higher in women than in men. This is consistent with the sex differences of endothelial function measured by flow-mediated dilation in healthy adults. However, the magnitude of the sex difference for VRI is not felt to be large enough to warrant establishing sex-specific cutoff values for good, intermediate, and poor vascular reactivity [30]. We also observed a trend of higher VRI values with higher diastolic BP but no association with systolic BP or pulse pressure. It is possible that the subjects with high BP would have been on multiple antihypertensive medications that could have increased their VRI scores. The results of multivariable analyses showed that SBP and DBP were found to have minimal correlations with age. Because blood pressure is known to correlate strongly with age in untreated population [31], BP-lowering medications may have played a significant role in modifying (leveling) the relationships in our study population. Because our data set was limited by the unknown status of BP medication, we were unable to investigate this further. The weak relationship between VRI and traditional risk factors was not unexpected.

Many investigators of peripheral vascular endothelial function refer to FMD as the method of choice and consider it to be a “reference” standard, but it has several physiological and technical issues, including operator-dependency, which may result in excessively high inter- and intraobserver variability, effect of the baseline diameter, low flow-mediated constriction, and reduced arterial wall compliance [32–34]. The correlation between FMD and finger-based measurements has been less than strong [7, 26, 35–38]. The weak or inconsistent correlations between FMD and the finger-based measurements have been explained by the notion that FMD mainly reflects macrovascular reactivity, whereas the finger-based measurements mostly reflect microvascular reactivity. Currently, there is no evidence regarding superiority of macrovascular over microvascular reactivity [39]. More studies are needed to evaluate the predictive value of each for risk assessment and monitoring response to therapies.

Previous studies showed a significant relationship between poor VRI and high Framingham Risk Score as well as high coronary calcium score [14, 18, 19]. Moreover, one study found that individuals with both poor VRI and high Framingham Risk Score had the highest coronary calcium scores [17]. Although more work is needed to develop clear clinical guidelines to incorporate such physiologic measurements into patient care, there is no doubt that these physiologic data offer a new window to an individualized assessment. The fact is that risk factors are population-based factors and do not speak for individual's susceptibility to the risk factors, nor can they evaluate the current status or activity level of the disease. On the other hand, structural markers such as coronary calcium and carotid IMT-plaque are good indicators of susceptibility to risk factors and show the effects of past exposure, but they do not show the current status or the activity level of the disease. In fact, calcification will not go away with treatments, making it not suitable for monitoring progression and regression. Measurement of endothelial function and vascular reactivity provides an instant status of the vascular physiology. Therefore, for a comprehensive assessment of vascular health, one must pay attention to risk factors, structural markers, and functional markers of the disease [1]. Although almost all CVD patients receive medications and other therapeutic interventions, not all respond to the treatments or respond similarly. Identifying who responds well and who responds poorly is a major challenge and currently classified as “residual risk.” Budoff et al. reported that VRI was significantly higher in patients who received statins and aged garlic extract, compared with those who received statin alone, and that patients who showed a significant improvement in VRI had less progression of coronary calcium [40]. Matsuzawa et al. showed an independent and significant predictive value for endothelial function measurement in patients at high risk for cardiovascular events [41]. Similarly, Rubinshtein et al. showed that poor fingertip-based vascular reactivity measurement with PAT significantly predicted poor outcomes. Together, these data clearly point to the clinical utility of endothelial function in primary and secondary prevention [42]. A detailed comparison of FMD, PAT, DTM, and other noninvasive CVD risk assessment methods is shown in Table 3.

## 5. Limitations

Several limitations of the current study must be considered. The primary limitation was that the VENDYS Registry data do not include information about patients' clinical conditions or medication use. We also did not know when the DTM tests were performed in relation to each individual's medical history and use of medications that might have affected vascular reactivity. Strengths of our study include a large sample size and a mixed population of males and females, geographically dispersed and from various outpatient clinics.

## 6. Conclusion

The present study using the largest database of finger-based assessment of endothelial function shows that digital thermal monitoring of vascular reactivity provides meaningful and reproducible physiological variables. It also suggests independent roles for VRI as new indices of vascular reactivity and endothelial function. DTM is very simple and inexpensive to perform. It is essentially a combination of a blood pressure and a thermometer empowered by intelligent software that can be used both at clinics and at home. However, further studies are needed to incorporate these functional measurements into clinical practice guidelines for primary and secondary prevention.

## Figures and Tables

**Table 1:** Selected patient and test characteristics.

Variable	Mean ± SD or % (*n*)
Age (y)	65.5 ± 13.7
Male/female	54%/46%
Systolic blood pressure (mmHg)	138 ± 20
Diastolic blood pressure (mmHg)	77 ± 12
Heart rate (bpm)	70 ± 13
Right finger *t*300 (°C)	32.1 ± 2.7
Left finger *t*300 (°C)	31.9 ± 2.8
Ambient temperature (°C)	24.3 ± 1.9
Cold Finger	5.8% (*n* = 353)
Sympathetic Response	4.8% (*n* = 294)
VRI score, overall	1.53 ± 0.53
VRI score, women	1.56 ± 0.58
VRI score, men	1.50 ± 0.49

Finger *t*300: finger temperature at the onset of cuff occlusion (time 300 s); VRI: vascular reactivity index; NVRI: neurovascular reactivity index; Cold Finger: a flagged condition in which right finger *t*300 is equal to or less than 27°C; Sympathetic Response: a flagged condition in which left finger temperature continuously declines after right arm-cuff occlusion.

**Table 2:** Multiple linear regression models for VRI, SBP, and DBP.

	*β*	*p* value
VRI (dependent) *R*-squared = 0.06, SE = 0.52		
Intercept	1.885539	<0.001
Age	−0.00826	<0.001
DBP	0.003341	0.002
Male sex	−0.09741	<0.001

SBP (dependent) *R*-squared = 0.02, SE = 20.36		
Intercept	119.3615425	<0.001
VRI	2.304075346	0.001
Age	0.186886935	<0.001
HR	0.067271865	0.018
Male sex	−0.412960283	0.560

DBP (dependent) *R*-squared = 0.10, SE = 11.78		
Intercept	70.26680855	<0.001
VRI	1.796063985	<0.001
Age	−0.150770023	<0.001
HR	0.1759533	<0.001
Male sex	3.509046088	<0.001

Results are shown for four separate multiple linear regression models: VRI (vascular reactivity index), SBP (systolic blood pressure), and DBP (diastolic blood pressure). *β* = *β* coefficient; *R*-squared: *R* ^2^; SE: standard error. Units for variables were as follows: age (y), HR (bpm), sex (male = 1; female = 0), SBP, and DBP (mmHg).

**Table 3:** Comparison between CVD risk assessment methods.

Method	Type (structural, functional, and risk factors)	Independent of age	Predictive value	Response to therapy	Ease of use and applicability in primary care setting	Intra- and interobserver reproducibility	Self-monitoring by patients at home
Coronary artery calcium	Structural	−	+++	−	+	+++	−

Carotid IMT and plaque	Structural	−	++	+	++	+	−

Ankle brachial index	Structural	−	++	−	+++	++	−

Arterial stiffness (e.g., PWV, AI, and *C*1/*C*2)	Structural/ functional	−	++	+	++	++	−

Risk factor-based risk calculators (e.g., FRS, SCORE, and QRISK2)	Risk factors	−	++	n/a	+++	++	++

FMD	Functional	+	++	++	−	−	−

PAT (RHI)	Functional	+	++	++	+++	++	++

PPG (RI)	Functional	+	++	++	+++	++	++

DTM (VRI)	Functional	+	++	++	+++	++	+++

Carotid IMT: carotid intimal-media thickness; PWV: pulse wave velocity; AI: augmentation index; *C*1/*C*2: indices of large and small artery compliance (elasticity); FRS: Framingham Risk Score; SCORE: Systematic Coronary Risk Evaluation risk score system published by the European Society of Cardiology; QRISK2: risk calculator developed by UK National Health Service; FMD: flow-mediated dilatation; PAT: peripheral arterial tonometry; RHI: reactive hyperemia index; PPG: photoplethysmography for digital pulse waveform analysis; RI: reflection index; DTM: digital thermal monitoring; VRI: vascular reactivity index.

## Additional Points

*Implications for Current Practice*. DTM is a noninvasive, automated, and operator-independent test that can be performed inexpensively. It is essentially a combination of a blood pressure and a thermometer empowered by intelligent software. It can be used both at physicians' offices and in patients' homes and it provides important physiologic insights into the functional aspect of vascular health. As a measure of endothelial function, it can be useful both in primary and in secondary prevention.* Futuristic Implications*. Empowering patients and healthcare consumers to monitor their health at home is the cornerstone of future healthcare. Preventive care does not belong to hospitals or physicians' offices; it belongs to home healthcare. Unfortunately, despite major technological advantages that revolutionized our life with personal digital devices (PDAs) and smartphones, personal healthcare devices do not yet exist. In fact, since the introduction of thermometers and blood pressure devices to home-based measurement many decades ago, very little has been done to empower self-monitoring at home. DTM can fill this gap. It is simple, easy-to-do, and operator-independent, all of which make it suitable as a home-based, self-administered test.
